# Knock, knock – who’s there? Doorway event boundaries weaken but do not eliminate bindings in first-person perspective environments

**DOI:** 10.3758/s13423-026-03036-w

**Published:** 2026-11-02

**Authors:** Maria Nemeth, Christian Frings, Christian Beste, Birte Moeller

**Affiliations:** 1 Department of Psychology, Cognitive Psychology, University of Trier, D-54286 Trier, Germany; 2 Department of Child and Adolescent Psychiatry, Faculty of Medicine, Cognitive Neurophysiology, University of Dresden, Dresden, Germany

**Keywords:** Event segmentation, Event boundaries, Action control, Event file, Response-response binding

## Abstract

Current theories of action control assume that the binding and retrieval of features into and from common memory representations (i.e., event files) constitute core mechanisms supporting goal-directed behavior. Recent work found that individual responses within ongoing action sequences can be bound as higher-order representations that persist long enough to influence hierarchical event perception and action control (e.g., Moeller & Frings, 2021; Weissman, 2025). Initial evidence suggests that these binding processes may be disrupted when event boundaries are encountered, yet it remains unclear whether boundaries invariably interfere with binding to the extent that the influence of previous actions on current ones (i.e., binding effects) is no longer measurable. To address this issue, we investigated response-response bindings in a first-person virtual scenario involving perceptually similar events that were separated by a spatial boundary (a doorway between rooms). Responses executed within a single event produced stronger binding effects than responses that spanned the event boundary, indicating that boundary perception can attenuate, but does not fully abolish, binding processes. These results suggest that the cognitive system organizes continuous perceptual streams into discrete events in a manner that modulates ongoing action control. Crucially, by demonstrating that binding processes adapt to, rather than fail at, naturalistic event transitions, the present results highlight a key mechanism that supports reliable yet flexible action control in everyday behavior.

## Introduction

In everyday life, human cognition is confronted with a continuous stream of information. To act adaptively, the cognitive system must organize this input into meaningful units. Research on event cognition proposes that experience is segmented into discrete events that structure perception and memory (Radvansky & Zacks, 2014; Tulving, 1972; Zacks & Swallow, 2007). In parallel, research on action control suggests that event files temporarily integrate features of stimuli, responses, and effects into episodic representations (Frings et al., 2020; Hommel, 1998, 2004). The present study connects these two lines of research by examining whether a subtle spatial event boundary modulates action control processes.

According to Event Segmentation Theory (EST; Zacks & Swallow, 2007), perceivers continuously compare perceptual input with predictions derived from event schemata. When incoming information deviates from expectations, the current event model is updated, resulting in an event boundary (Richmond & Zacks, 2017; Zacks, 2020). The Event Indexing Model further proposes that changes in dimensions such as space, time, goals, causality, or protagonists trigger updating of event representations (Zwaan et al., 1995; Zwaan et al., 1998; see also Zacks, 2020). Event boundaries lead to memory links being weakened, and information encountered just before a boundary becoming less likely to be retrieved subsequently (DuBrow & Davachi, 2016; Speer & Zacks, 2005; for neurophysiological evidence, see Prochnow et al., 2024; Zheng et al., 2022).

Empirically, event boundaries are assessed by asking observers to segment ongoing activity into meaningful units (Newtson, 1973; Newtson et al., 1977), and these segments reliably correspond to memory representations (Ezzyat & Davachi, 2011; Huff et al., 2014; Pettijohn et al., 2016). Importantly, although EST specifies when event models are updated, it remains largely agnostic about the magnitude of this updating, leaving open whether event boundaries produce graded changes in ongoing representations or more abrupt reconfigurations.

A prominent demonstration of boundary effects on memory is the location-updating effect, in which participants encode objects while navigating an environment and subsequently show reduced memory for task-relevant information after passing through a doorway (Radvansky & Copeland, 2006). This finding has been replicated across real and virtual environments as well as active and passive first-person tasks (Pettijohn & Radvansky, 2016a, b, 2018; Radvansky et al., 2010, 2011), suggesting that task-irrelevant spatial boundaries are particularly potent segmentation cues that reorganize episodic memory representations.

Action-control research provides a complementary account of how recent experiences influence ongoing behavior (Frings & Beste, 2026). According to contemporary ideomotor approaches, features of stimuli, responses, and action effects are temporarily integrated into event files (Beste et al., 2023; Frings et al., 2020; Hommel, 1998, 2004). Re-encountering event file features later retrieves the bound features, including the previous action.

Recent work suggests that event boundaries can eliminate the influence of binding and retrieval processes. Moeller et al. (2025) observed no response-response binding effects when responses had to be either bound (Experiment 1) or retrieved (Experiment 2) across an event boundary. In their task, participants either maneuvered a monkey in a jungle or a fish underwater, and event boundaries were induced by switching between these two characters and environments, hence highly salient. Converging evidence comes from Qiu et al. (2023), who reported an absence of binding effects, when information was to be retrieved across a different sound context. Together, these findings thus far suggest that event boundaries can fully “reset” the memory-based influence of prior actions, at least when boundaries are highly salient.

However, this interpretation is primarily based on paradigms employing highly salient, multi-dimensional boundaries (e.g., changes in agent, environment, and task context). Although salient environmental changes especially in visual input are widely assumed to guide the location of event boundaries (e.g., Hard et al., 2011; Zacks et al., 2009), natural environments are not always marked by such dramatic transitions. Instead, boundaries may also be triggered by more moderate spatial cues, such as passing through a doorway, even when adjoining scenes remain perceptually similar. Although doorway crossings reliably influence episodic memory, it remains unknown whether they affect binding and retrieval in the same way as the highly salient boundaries used previously.

Addressing this question is theoretically important because current accounts do not specify how the strength or type of an event boundary determines its influence on action control. Existing findings clearly demonstrate that highly salient boundaries can abolish binding and retrieval (Moeller et al., 2025; Qiu et al., 2023), whereas work on event segmentation more generally suggests that event boundaries reduce accessibility of prior information without necessarily eliminating all cross-boundary influences (DuBrow & Davachi, 2016; Richmond & Zacks, 2017). Consequently, subtle spatial boundaries may either substantially disrupt binding and retrieval or merely attenuate these processes, allowing previous actions to continue influencing behavior across event segments. Determining which of these possibilities better characterizes more subtle boundaries informs how flexibly action control adapts to the structure of ongoing experience.

### The present study

**Fig. 1:**
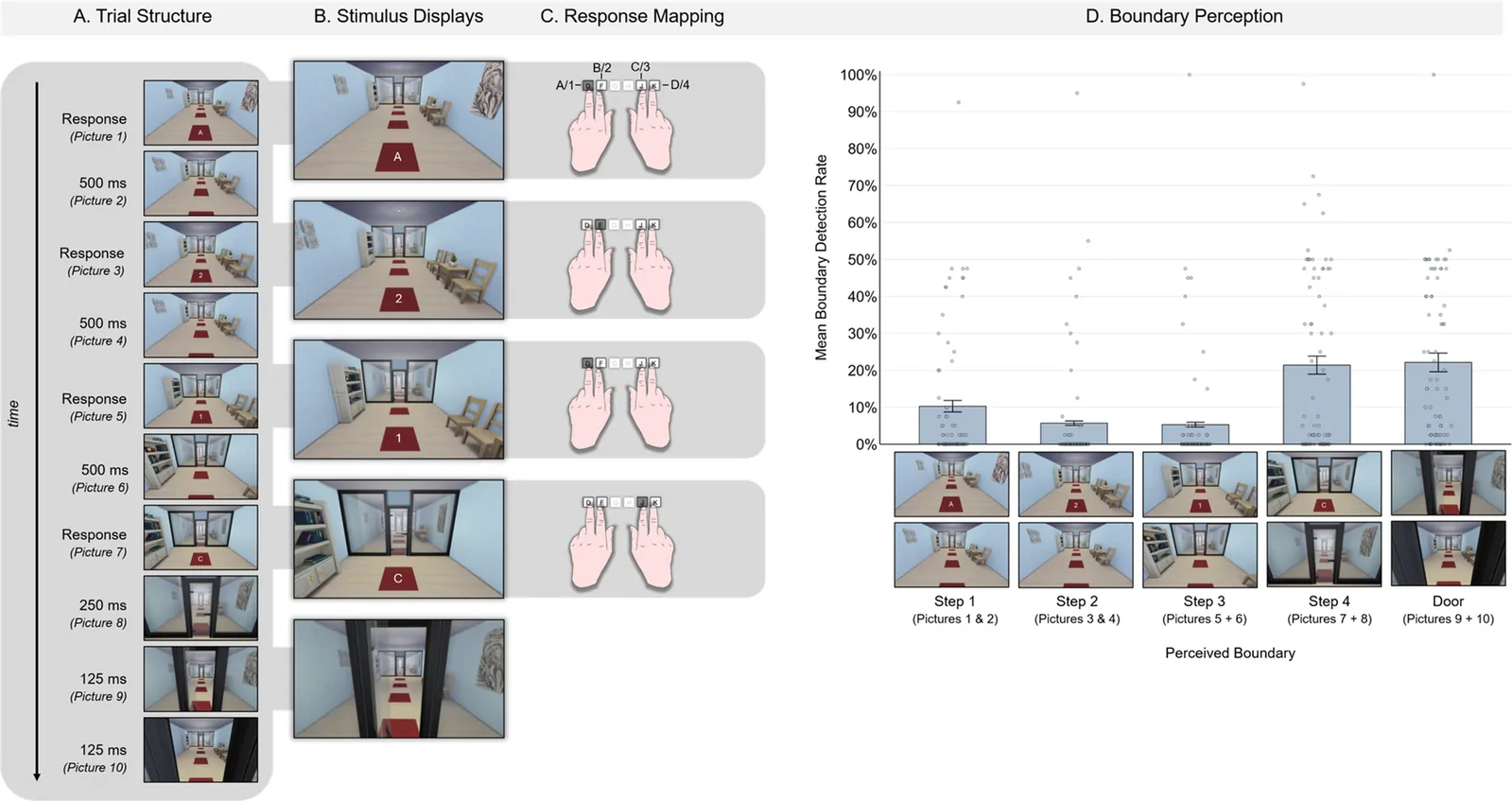
Experimental set-up and manipulation check. **A**–**C** Participants viewed picture sequences depicting movements through a series of connected rooms via a door. Participants responded with their index and middle fingers to the identity of individually presented digit and letter stimuli. Each response advanced the perspective by one step within the virtual hallway. After four responses in one scenario and entering through the door into the next room, the scenario switched into the next room starting from the initial position. Spatial location changes (doorways) were task irrelevant and served as incidental event boundary cues. The response-stimulus interval was always 500 ms. This is an example of a Response R1 repetition and Response R2 change trial. **D** Boundary detection rate in percentages in the manipulation check. Error bars indicate ±1 standard errors of the mean

The present study investigated whether more subtle event boundaries disrupt binding and retrieval (see Fig. 1). Specifically, we asked whether crossing a doorway in a first-person perspective scenario, serving as a spatial boundary between otherwise perceptually similar scenes, diminishes the influence of previous actions on current ones via response binding and retrieval. Importantly, spatial location changes (doorway crossings) were task-irrelevant and served as incidental event boundary cues, analogous to location-updating paradigms (Radvansky & Copeland, 2006), in which spatial changes are not explicitly attended but still trigger event model updating.

A standard response-response binding paradigm was used (Moeller & Frings, 2019), in which participants responded to two sequentially presented stimuli in a prime episode, followed by two responses in a probe episode. Executing the two prime responses is assumed to lead to their binding. Subsequent repetition of the first prime response in probe can then trigger retrieval of the previously bound prime response, such that performance is facilitated when the retrieved response matches the currently required probe response but is impaired if it mismatches. Accordingly, response-response binding is indexed by the interaction between the relation of the first response and the second response from prime to probe.

Crucially, we compared binding effects when a spatial boundary occurred between the two to-be-bound responses with a control condition in which the boundary occurred before these responses. Prior work on location updating suggests that crossing a doorway can constitute a meaningful event boundary, even when visual input remains largely similar (Pettijohn & Radvansky, 2016a; Experiment 3). Thus, the key question was whether a boundary defined primarily by spatial change, without substantial shifts in scene content or acting agent, is sufficient to disrupt binding of responses and their retrieval. If such a spatial boundary between two responses were sufficient to eliminate binding, this would suggest that event boundaries could impose a general constraint on action control processes, limiting the influence of recent action episodes whenever a boundary is encountered. Conversely, if binding and retrieval effects persist (although potentially reduced) despite a boundary occurring between responses, this would indicate that the cognitive system adapts flexibly and with fine-grained sensitivity to the structure of the environment, allowing continuity in action control across less salient event boundaries.

## Methods

### Transparency and openness

We report how we determined our sample size, all data exclusions, all manipulations, and all measures in the study. The study was not preregistered. The study was conducted in accordance with the ethical guidelines declared by the Ethics Committee of the University. The Ethics Committee of the University declares all simple behavioral studies in accordance with their guidelines exempt from any further examinations by the committee. Raw data of the reported experiments can be found on the Open Science Framework (OSF) (https://osf.io/vpyjh/overview?view_only=190d8d91a5a44d409034bdb93dd7153e).

### Participants

The sample size was based on the study by Moeller et al. (2025), who also investigated the influence of event boundaries on the binding between responses. In their study, 39 participants were tested. As Moeller et al. (2025) employed event boundaries across multiple dimensions, whereas the present study focused on a spatial boundary manipulation, we aimed to detect at least a small-to-medium effect for differences in binding across event segments. On this basis, we aimed to approximately double the sample size. Seventy-six participants from the University participated in the experiment (55 female, 20 male, one not specified) with a median age of 23 years (range 18–44 years). All participants signed a written informed consent form and received course credit for their participation.

### Design

The design was adapted from previous studies, investigating response-response bindings (e.g., Moeller et al., 2025; Moeller & Frings, 2021). It comprised three within-subjects factors: Response R1 relation from prime to probe (response repetition vs. response change), Response R2 relation from prime to probe (response repetition vs. response change), and event boundary (before to-be-integrated responses vs. between to-be-integrated responses).

### Apparatus, stimuli, and basic paradigm

The experiment was programmed in PsychoPy (2023.2.3; Peirce et al., 2019) and participants conducted the experiment in the university’s laboratory on standard laboratory computers. Instructions were shown in white (font: Arial; font size: 20 pixels) on a gray background. Backgrounds during the experiment were virtual rooms that were separated by a door. Stimuli were the digits 1, 2, 3, and 4 and the letters A, B, C, and D. All digits and letters were presented in white (font: Arial; Font size: 40 pixels). In the experimental phase, participants responded by pressing one of four keys (D, F, J, or K) on a standard German QWERTZ keyboard. In the event-segmentation phase, participants responded by pressing the space bar.

### Procedure

Participants were presented with written instructions of the experimental procedure on the screen. Participants were instructed to place the middle and index fingers of both hands on the keys D, F, J, and K of their keyboard. They were instructed that their left middle finger was mapped to the stimuli A and 1, their left index finger to the stimuli B and 2, their right index finger to the stimuli C and 3, and their right middle finger to the stimuli D and 4. Participants were instructed to press the key corresponding to individually presented digits and letters. The virtual environment consisted of rooms containing four rectangular carpets, arranged vertically so that their long axes pointed toward a door within each room. Participants were told that they would move straight ahead through the rooms by responding to the stimuli, thereby “clearing” the letters and digits from their path (see the OSF repository for a short video demonstration of the experimental procedure from the participant’s perspective). They were encouraged to perform the task as quickly as possible while maintaining a high level of accuracy.

Before starting the experimental block, participants completed 16 training trials. In training, participants received performance-contingent feedback after each response (for a correct response: “correct!”; for a wrong response: “wrong!“). In the experimental block, feedback was only provided for incorrect answers. There was no maximum time for the responses; the stimuli and respective background remained on the screen until participants responded.

**Fig. 2:**
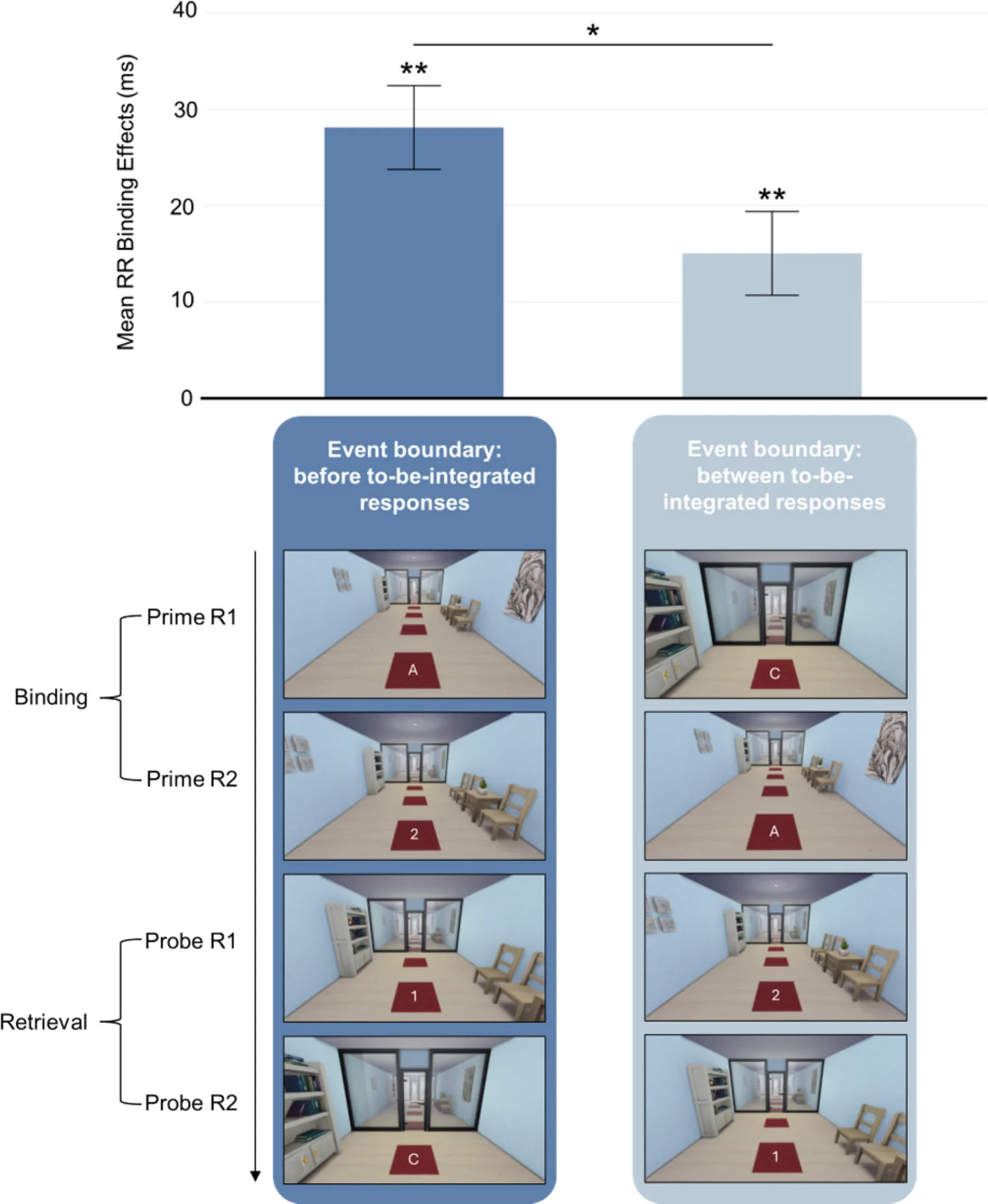
Individual response-response binding effects as a function of whether to-be integrated responses were given within or across event boundaries (i.e., doorways). In the event boundary condition before to-be-integrated responses, prime responses R1 and R2 were given in the same virtual rooms. In the event boundary condition between to-be-integrated responses, prime response R1 was given in one room, followed by a transition through the door, where prime response R2 was executed. R-R= response-response. Error bars indicate ±1 standard errors of the mean. **p <.010, *p <.050

The experimental block comprised two blocks. A single trial required participants to execute four individual responses (prime R1, prime R2, probe R1, probe R2) in the following sequence of events (see Fig. 1). In the block with the event boundary *before to-be-integrated responses*, all four responses of one trial were performed within the same virtual room (see Fig. 2). In the beginning of the trial, the prime R1 display showed four rectangular carpets vertically arranged on the floor, oriented toward a door.^1^ The prime R1 stimulus appeared before the background of the scenery on top of the first carpet. After participants responded, the stimulus disappeared, and only the rear half of that carpet remained visible, positioned closer to the next carpet in the sequence and giving the impression of having moved forward in the room towards the door through responding. After 500 ms, the prime R2 stimulus appeared on the next carpet. Responding again caused the stimulus to vanish and the corresponding carpet to be partially concealed. After 500 ms, the probe R1 stimulus was presented on the third carpet. Responding again triggered the same visual “step forward,” and after 500 ms the probe R2 stimulus appeared on the final carpet indicating the last response of the trial. After participants responded, the stimulus disappeared, and only the rear half of that carpet remained visible, positioned closer to the doorway (125 ms). In the subsequent display, the viewpoint advanced further so that the scene was shown from a location within the doorway for 125 ms, giving the impression of passing through the door. Then the prime R1 stimulus was again presented on the very first of four carpets in the same room. In case of an incorrect response, an error message was presented in the center of the screen (before the background of the scenery) for 1,000 ms immediately following the erroneous response.

In a second block with the event boundary *between to-be-integrated responses*, participants experienced the same sequence of events. However, here the prime R1 response as a first response was to be made towards a stimulus that was presented on the very last carpet before the door, and the three remaining responses were made towards the first three carpets in the new room (see Fig. 2). Thus, the door as the event boundary after the prime R1 response served for us to measure binding and retrieval effects in probe R2 responses, after prime R1 and prime R2 responses had to be integrated across the event boundary.

The factors Response R1 relation of response R1 between prime and probe (repetition vs. change) and Response R2 relation between prime and probe (repetition vs. change) were varied orthogonally. In R1 repetition trials, the same response required in prime R1 was also required in probe R1. In R1 change trials, a different response was required in prime R1 and probe R1. In R2 repetition trials, the identical response required in prime R2 was also required in probe R2. In R2 change trials, a different response was required in prime R2 and probe R2. At the beginning of each trial, the prime R1 stimulus was randomly drawn from the stimulus array. The corresponding probe R1 stimulus was then selected according to the experimentally specified R1 repetition or change relation. The prime R2 stimulus was subsequently randomly selected, followed by selection of the probe R2 stimulus according to the specified R2 repetition or change relation. Thus, stimulus selection was randomized within the constraints imposed by the experimental response relations, and for each response, whether a letter or a digit served as stimulus was randomly assigned. Importantly, stimuli did not repeat between prime and probe, thus a response repetition from prime to probe always involved a change from a letter to a digit or vice versa.

The factor event boundary (before first to-be-integrated response vs. between to-be-integrated responses) was varied blockwise. The order of blocks was counterbalanced across participants (note that block order did not affect any relevant effect^2^). Each experimental block included 128 trials (32 of each of the four conditions R1 repetition R2 repetition, R1 repetition R2 change, R1 change R2 repetition, R1 change R2 change). The order of blocks was counterbalanced across participants.

At the end of the experiment, participants completed a manipulation check assessing the perception of event boundaries in the current task. They were informed that they would again see the pictures from the experiment, presented one after another, and were instructed to press the space bar whenever they perceived that one meaningful unit had ended and another had begun. Each picture was presented for 1,000 ms, and the entire sequence was repeated 20 times.

### Manipulation check

In order to examine whether the boundary manipulation was successful, we analyzed whether participants indicated the doorway boundary as such in the manipulation check phase. For ease of interpretation, the ten possible boundary positions were collapsed into five grouped steps: *step 1* (four steps to door and half step 1), *step 2* (three steps to door and half step 2), *step 3* (two steps to door and half step 3), *step 4* (one step to door, closed door), and *door* (front door, and through door).

A repeated-measures ANOVA with mean boundary detection rate as a dependent variable revealed that the main effect of boundary position was significant, *F*(1,296) = 22.11, *p* <.001, *η*_p_^2^ =.13. Helmert contrast coding revealed that the mean boundary detection rate for Door was higher as compared to the mean of all other conditions (*t*(296) = 5.83, *p* <.001), that the mean boundary detection rate for Step 4 was higher as compared to the mean boundary detection for Step 1, Step 2, and Step 3 (*t*(296) = −2.16, *p* =.032), that there was no difference in mean boundary detection rate for Step 3 compared to the mean boundary detection for Step 1 and Step 2 (*t*(296) = −1.25, *p* =.215), and that there was no difference in mean boundary detection rate for Step 2 and Step 1 (*t*(296) = −1.83, *p* =.068). Thus overall, boundary detection rates increased as the sequence approached the doorway, consistent with the intended event-segmentation manipulation (see Fig. 1).^3^

## Results

All data processing and analysis were conducted in R (R Core Team, 2019; R Version 4.2.2). The response-response binding effects for both reaction times and error rates were calculated as the advantage of probe R1 repetition over probe R1 change in probe R2 repetition trials minus the advantage of probe R1 repetition over probe R1 change in probe R2 change trials: (R1cR2r-R1rR2r)-(R1cR2c-R1rR2c).

### Analysis of experimental hypotheses

This section presents the analyses testing the main experimental hypotheses concerning the modulation of response-response binding effects by event boundaries. Performance in probe R2 was the dependent variable of interest. If the two prime responses R1 and R2 were integrated, repeating prime response R1 as probe R1 may trigger retrieval of prime R2, which would then influence probe R2 performance. Only trials with correct responses in both prime and probe were considered for the analysis of probe R2 RTs. The rate for errors in prime R1 was 5.12% and for errors in prime R2 5.6%. Probe error rates were 4.9% for R1, and 5.2% for R2 (only including trials without errors in the previous responses). In addition, trials with probe R2 reaction time (RT) below 200 ms and more than 1.5 interquartile ranges above the third quartile of each subject’s RT distribution were excluded (see Tukey, 1977). Due to these constraints, a total of 23.0% of all trials were excluded from analyses (see Table 1 for mean RTs and Table 2 for error rates).

**Table 1:** Mean reaction times (RTs) in ms (*SD*s in parentheses) for probe response R2, as a function of Response R1 relation from prime to probe (repetition vs. change), Response R2 relation from prime to probe (repetition vs. change), and event boundary (before to-be-integrated responses vs. between to-be-integrated responses)

	Before to-be-integrated responses		Between to-be-integrated responses	
	R2 repetition	R2 change	R2 repetition	R2 change
R1 repetition	555 (105)	561 (111)	565 (107)	569 (109)
R1 change	573 (111)	552 (108)	575 (117)	563 (113)

**Table 2:** Mean error rates in percentages (*SD*s in parentheses) for probe response R2, as a function of Response R1 relation from prime to probe (repetition vs. change), Response R2 relation from prime to probe (repetition vs. change), and event boundary (before to-be-integrated responses vs. between to-be-integrated responses)

	Before to-be-integrated responses		Between to-be-integrated responses	
	R2 repetition	R2 change	R2 repetition	R2 change
R1 repetition	4.6 (3.6)	4.5 (4.2)	5.6 (5.5)	5.0 (5.0)
R1 change	6.7 (5.9)	5.0 (5.2)	6.6 (5.8)	5.5 (4.9)

A 2 (within: Response R1 relation: repetition vs. change) × 2 (within: Response R2 relation: repetition vs. change) × 2 (within: event boundary: before to-be-integrated responses vs. between to-be-integrated responses) ANOVA on probe R2 RTs was conducted. The main effect of Response R2 relation was significant, *F*(1,74) = 11.49, *p* =.001, *η*_p_^2^ =.13. Participants responded slower if R2 repeated (*M* = 567 ms, *SD* = 110 ms) than if R2 changed from prime to probe (*M* = 561 ms, *SD* = 110 ms). The main effect of event boundary was significant, *F*(1,74) = 4.82, *p* =.031, *η*_p_^2^ =.06. Participants responded faster if the event boundary was before prime responses (*M* = 560 ms, *SD* = 108 ms) than between prime responses (*M* = 568 ms, *SD* = 111 ms). The interaction of R1 relation with R2 relation was significant, *F*(1, 74) = 43.73, *p* <.001, *η*_p_^2^ =.37, indicating binding between the responses. Importantly, this response-response binding effect was modulated by event boundary, *F*(1, 74) = 4.52, *p =*.037, *η*_p_^2^ =.06. Post hoc *t* tests revealed a stronger response-response binding effect in the before to-be-integrated responses condition (*M* = 28 ms, *SD* = 41 ms; *t*(74) = 5.95, *p* <.001, *d* = 0.69) than in the between to-be-integrated responses condition (*M* = 15 ms, *SD* = 37 ms; *t*(74) = 3.56, *p* <.001, *d* = 0.41), *t*(74) = 3.56, *p* =.037, *d* = 0.25 (see Fig. 2).^4^ None of the other effects reached significance; all *F* < 3, *p* >.13, *η*_p_^2^ <.03.

A post hoc power analysis was conducted G*Power (Faul et al., 2007) to assess the statistical power of the critical *t*-test comparison. Based on an observed effect size of *d* = 0.25, an alpha level of α = 0.05, and a sample size of *N* = 76, the power was calculated to be 1˗β = 0.58, indicating that there was a 58% chance of detecting an effect of this size. Furthermore, a sensitivity analysis was conducted to determine the smallest detectable effect size with a sample size of 76, an alpha level of α = 0.05, and a desired power of 0.80: The analysis indicated that the experiment was powered to detect an effect size of at least *d* = 0.33.

In the same analysis on probe R2 error rates the numerical pattern was similar. However, only the main effect of Response R1 relation was significant, *F*(1,74) = 8.52, *p* =.005, *η*_p_^2^ =.10. Participants made fewer errors if R1 repeated (*M* = 4.9%, *SD* = 4.6%) than if R1 changed from prime to probe (*M* = 5.9%, *SD* = 5.5%). All other effects were *F* < 4, *p* >.06, *η*_p_^2^ <.05.

## Discussion

The present study investigated how perceived environmental structure influences the formation of common representations of ongoing action sequences and how these representations subsequently influence actions. Specifically, we examined whether response-response binding is modulated by subtle event boundaries induced by a doorway transition. Three main findings emerged. First, the door was more consistently perceived as an event boundary compared to the other scenes. Second, response-response binding effects were reduced when the boundary occurred between the two to be integrated responses compared to when it preceded them. Crucially, however, binding effects remained reliably observable even when responses were separated by a boundary. Together, these results demonstrate that event boundaries modulate binding and retrieval processes but do not necessarily abolish them.

### Theoretical implications

The present study provides novel evidence for the robustness of binding mechanisms under conditions of event segmentation. In contrast to previous findings suggesting that event boundaries completely disrupt binding (Moeller et al., 2025; Qiu et al., 2023), the current results show that bindings can persist even when the features to be integrated (i.e., successive responses) are separated by a spatial boundary. These findings support a view in which event boundaries scale, rather than generally eliminate, action control mechanisms. Specifically, rather than abolishing feature integration across events, the cognitive system appears capable of forming higher-order representations that connect successive events despite boundaries. This continuity is likely adaptive in everyday behavior, where actions often unfold across subtle transitions that do not require a complete updating of internal models or event files. For instance, when navigating a building, opening a door may signal a new context, yet ongoing higher-order action plans such as walking toward a destination and preparing to interact with an object are able to be maintained. Preserving bindings across such transitions allows recently executed actions to be efficiently reused, thereby supporting coherent and goal-directed behavior.

At the same time, an important consideration concerns the effective strength of the boundary manipulation. Although participants identified the doorway position as an event boundary more frequently than other positions, detection rates were overall moderate, suggesting that the doorway constituted a relatively subtle segmentation cue. Accordingly, the observed attenuation of binding effects may partly reflect the limited strength and consistency with which the doorway was perceived as an event boundary. Importantly, this interpretation does not challenge our general conclusion that event boundaries attenuate binding but rather qualifies its scope by suggesting that the magnitude of this attenuation may depend on boundary strength. This interpretation also fits with the result that explicit boundary judgments did not predict the degree of response-response binding attenuation (as indicated in the regression analysis). Given the moderate boundary judgments, this null finding should not be interpreted as evidence against a role of boundary strength – rather it highlights the need for studies that manipulate boundary strength directly as observing a significant correlation requires reliable measurements of individual boundary judgements and individual binding attenuation; this study was planned with measuring these variables at the group level though.

The present findings suggest that detecting an event boundary does not necessarily preclude binding formation, but that bindings can span segmented events under conditions in which a full representational reset is not required. This interpretation aligns with the assumption that event segmentation operates in a graded manner. While some boundaries primarily induce fine-grained segmentation, others trigger updates of coarser event models, which should have stronger effects on memory retrieval (Zacks, 2020). In fact, boundary perception is assumed to depend on the additive and interactive influence of multiple contextual dimensions (Magliano et al., 2001; Rapp & Taylor, 2004; Rinck & Weber, 2003; Zwaan et al., 1998). In the present study, the boundary was defined exclusively on the spatial dimension and was not accompanied by temporal, causal, or goal-related shifts. The observed reduction but not elimination of binding effects is therefore consistent with the possibility that both event boundary perception and its impact on action control increase with the number and relevance of changing dimensions.

## Limitations and future directions

Since binding and retrieval constitute dissociable processes, manipulations applied during either the integration or retrieval phase are typically interpreted as primarily affecting binding or retrieval, respectively (Frings et al., 2020). In the present study, however, the event boundary between prime R1 and prime R2 could in principle have affected both processes: it separated the responses to be integrated, potentially weakening their binding, while also placing subsequent retrieval of prime R1 in a different event context. Thus, in the present study, reduced response-response binding effects across the boundary may reflect weaker binding, reduced retrieval, or both. This consideration also applies to Moeller et al. (2025, Experiment 1). In contrast, when the manipulation targets the retrieval-triggering response (Moeller et al., 2025, Experiment 2), it can be assumed to primarily affect retrieval processes without directly influencing binding. Given that the present study focused on binding formation across event boundaries, a direct manipulation of retrieval was not implemented.

More generally, conclusions regarding graded influences of event boundaries on binding processes remain indirect, as boundary strength was not manipulated within the present design. A more direct test of graded boundary strength effects will require systematic manipulation of boundary salience. Future work could compare subtle spatial transitions, such as doorway crossings, with highly salient boundaries involving simultaneous changes across multiple event dimensions or parametrically vary the number of boundary-defining features within a single paradigm. Such approaches would allow a more fine-grained assessment of whether increasing boundary strength leads to progressively stronger modulation of response-response binding.

Finally, although the spatial boundary was experimentally controlled, it remains an artificial manipulation embedded in a simplified task environment. Early event segmentation studies relied on naturalistic activities without explicit boundary manipulations (e.g., observing building a tent; Zacks & Swallow, 2007). Combining such naturalistic observational paradigms with measures of action control represents a promising direction for future research, allowing observer-identified boundaries in real-world behavior to be linked directly to binding and retrieval processes.

## Data Availability

The datasets generated and/or analyzed during the current study are available in the OSF repository [https://osf.io/vpyjh/overview?view_only=190d8d91a5a44d409034bdb93dd7153e]. None of the experiments were preregistered.

## Footnotes

1 In addition to the event boundary manipulation, we varied the opacity of the doorway (transparent vs. opaque) to test whether visual access to the adjacent room modulates the perceived strength of the boundary and its effects on response-response binding. This factor did not interact with any theoretically relevant effects and is therefore not discussed further (see Pettijohn & Radvansky, 2016a).

2
*Block Order A*: (1) before to-be-integrated responses, (2) between to-be-integrated responses; *Block Order B*: (1) between to-be-integrated responses, (2) before to-be-integrated responses

3 Note that the Helmert contrasts did not directly test the difference between Step 4 and Door. Moreover, because Step 4 comprised both the scene immediately preceding the door and the closed-door scene, the analysis does not distinguish whether boundaries were perceived at either of these individual positions.

4 As an additional analysis, we examined whether individual differences in boundary perception predicted the modulation of response-response binding effects. For each participant, we computed the proportion of doorway scenarios classified as event boundaries (boundary detection rate) and regressed the reduction in the binding effect on boundary detection rate, boundary condition, and their interaction. The overall model was not significant, *F*(9, 360) = 0.35, *p* =.957, *R*^2^ =.009. Neither the main effect of boundary detection rate, *b* = 40.26, *p* =.324, nor its interaction with the Door condition, *b* = −43.97, *p* =.396, reached significance, indicating that explicit boundary judgments were not associated with the magnitude of binding modulation in the present experiment.
